# Electrospun Solid Formulation of Anaerobic Gut Microbiome Bacteria

**DOI:** 10.1208/s12249-020-01769-y

**Published:** 2020-07-31

**Authors:** Panna Vass, Eszter Pantea, András Domokos, Edit Hirsch, Júlia Domján, Áron Németh, Mónika Molnár, Csaba Fehér, Sune K. Andersen, Tamás Vigh, Geert Verreck, István Csontos, György Marosi, Zsombor K. Nagy

**Affiliations:** 1 Department of Organic Chemistry and Technology, Budapest University of Technology and Economics (BME), Műegyetem rakpart 3, Budapest, H-1111 Hungary; 2 Department of Applied Biotechnology and Food Science, Budapest University of Technology and Economics (BME), Műegyetem rakpart 3, Budapest, H-1111 Hungary; 3 Oral Solids Development, Janssen R&D, Turnhoutseweg 30, B-2340 Beerse, Belgium

**Keywords:** aqueous electrospinning, scaled-up production, cyclodextrin, bacteria-loaded fibers, oral dosage form

## Abstract

A model anaerobic bacterium strain from the gut microbiome (*Clostridium butyricum*) producing anti-inflammatory molecules was incorporated into polymer-free fibers of a water-soluble cyclodextrin matrix (HP-β-CD) using a promising scaled-up nanotechnology, high-speed electrospinning. A long-term stability study was also carried out on the bacteria in the fibers. Effect of storage conditions (temperature, presence of oxygen) and growth conditions on the bacterial viability in the fibers was investigated. The viability of the sporulated anaerobic bacteria in the fibers was maintained during 12 months of room temperature storage in the presence of oxygen. Direct compression was used to prepare tablets from the produced bacteria-containing fibers after milling (using an oscillating mill) and mixing with tableting excipients, making easy oral administration of the bacteria possible. No significant decrease was observed in bacterial viability following the processing of the fibers (milling and tableting).

## INTRODUCTION

Since the start of the Human Microbiome Project in 2008, it has become evident that the microbes that live in the human body—called the microbiome—play critical roles in biological processes, including intestinal homeostasis, metabolism, and development of the immune system, among others (1). Composition changes in the intestinal microbiota have been correlated to diverse, complex diseases including metabolic diseases (diabetes, obesity), inflammatory bowel disease, allergy, asthma, cancer, and neurologic and cardiovascular diseases (2). Based on these links, numerous pharmaceutical companies started to focus on the development of microbiome therapeutics (3,4). Defined bacterial consortia—generally consisting of 2 to 20 different bacterial strains—have been shown to enable the tailored manipulation of the microbiome, making it possible to use them as effective biotherapeutics (5,6). The manufacturing of such drugs is a great challenge since fermentation and solid formulation needs to be developed separately for each strain, which means that the development times and costs are multiplied by the number of strains in the given consortium. In addition, the gut microbiome is dominated by obligate anaerobic bacterial strains making the development of microbiome-based therapeutics even more challenging (7).

**Fig. 1:**
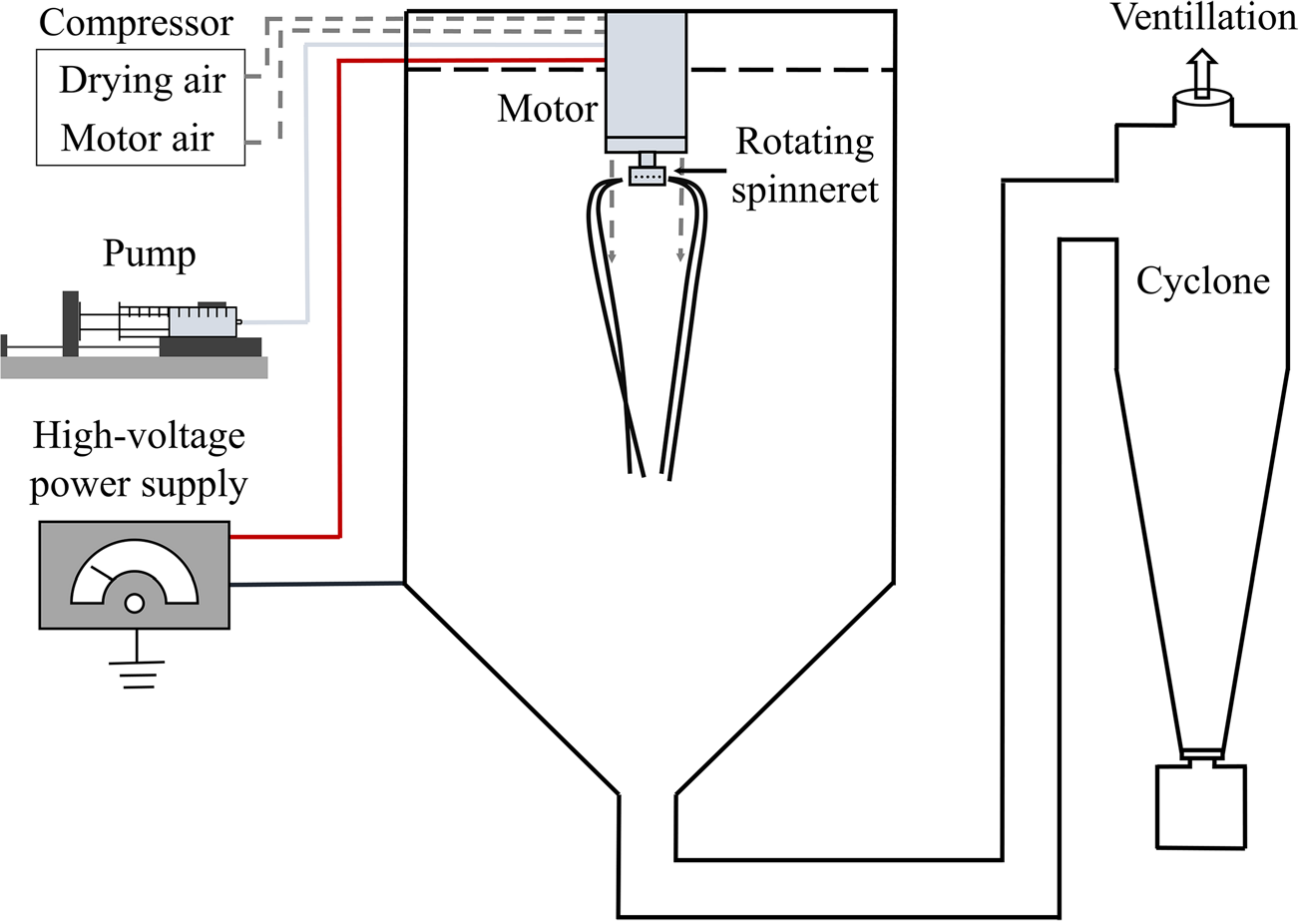
Schematic drawing of the high-speed electrospinning system connected to a cyclone

Today’s microbiome-based therapeutic agents are formulated using freeze drying despite the disadvantages of the technology. Freezing stresses caused by freeze drying can lead to the loss of bacterial viability. In addition, freeze drying is a batch technology with high energy, cost, and time demands. Testing a single set of process parameters takes from days to a week, which can make formulation development extremely lengthy, especially in the case of multiple bacterial strains. In contrast, electrospinning (ES) is an instant (drying time < 1 s) and efficient continuous drying method, which enables the screening of a large number of process conditions and formulation compositions in a single day. The most exciting feature of electrospinning for biopharmaceuticals is its ability to create nanostructured fibers (fiber diameters generally < 20 μm) from highly viscous solutions at room temperature (8). The technology generates stretched ultra-fine liquid jets from the liquid feed by electrical forces (9). The solvent evaporates extremely rapidly at room temperature due to the large surface area of the produced jets (10). Electrospinning is expanding its capability of creating functional fibers along several directions: scaled-up production (11,12), controlled release of the active pharmaceutical ingredient (API) (13), loading new active ingredients (14,15), application of non-polymer filament-forming matrices (16), creating complex nanostructures (17–19), and new knowledge on the working processes (20). In this work, several of the aforementioned novel directions were combined. A recently developed pharmaceutical industry compatible scaled-up device applying high-speed electrospinning (HSES) (Fig. 1) was used for the mass production of the bacteria-loaded fibers (21). By combining electrostatic (22) and high-speed rotational (23) jet generation and fiber elongation, the productivity of HSES is substantially increased compared with the productivity of the traditionally used technology with a single needle. Most of the previously developed high-throughput electrospinning technologies were free surface techniques immersing a cylinder (24) or a ball (25) into the electrospinning solution or covering a wire (26) with solution. However, the large free liquid surface usually leads to the evaporation of volatile solvents, which results in a constantly changing concentration in the liquid film making the process challenging to control (12). Solvent evaporation is minimized in HSES as free solution surface is eliminated by employing a rotating spinneret with orifices. HSES is a high-yield drying technology with low energy consumption that can be operated continuously, which make it an economical alternative to freeze drying. In previous works, water-soluble cyclodextrins were successfully electrospun (27–29), and therefore, in this work, hydroxypropyl-beta-cyclodextrin was selected as a promising non-polymeric matrix for the drying of this new bio-API.

Recently, laboratory-scaled electrospinning has been demonstrated to be a suitable solid formulation technology for various bacterial strains (30–37). Microbiome-based drugs usually target the colon, and therefore, they are most suitably administered orally in the form of tablets or capsules. However, according to the authors’ best knowledge, no attempt has been made yet to develop an oral final dosage form from bacteria-containing electrospun fibers. Therefore, two goals of this study were set: first, to demonstrate that the scaled-up production of bacteria-containing fibers with long-term stability is possible by high-speed electrospinning at room temperature and, secondly, to prepare tablets from the produced bacteria-loaded fibers. The model bacterial strain used in this work was *Clostridium butyricum*, which are strictly anaerobic spore-forming bacteria normally found in the human gastrointestinal tract (38). *C. butyricum* has been shown to regulate gut homeostasis and anti-inflammatory response in inflammatory bowel disease by the production of butyrate, which makes it a good model for the research of microbiome-based therapeutics (39).

## MATERIALS AND METHODS

### Materials

Hydroxypropyl-beta-cyclodextrin (HP-β-CD) (Kleptose® HPB, MS = 0.62) was obtained from Roquette Pharma (Lestrem, France). *Clostridium butyricum* Prazmowski 1880 was obtained from the National Collection of Agricultural and Industrial Microorganisms (Budapest, Hungary). Clostridial nutrient medium (CNM), tryptic soy broth (TSB), and tryptic soy agar (TSA) were purchased from Merck KGaA (Darmstadt, Germany). Columbia blood agar plates were obtained from Biolab Zrt. (Budapest, Hungary). AnaeroGen™ 2.5 L Sachets (anaerobic gas generating sachets) were purchased from Diagon Ltd. (Budapest, Hungary). Microcrystalline cellulose (MCC) (Vivapur 200) was purchased from JRS Pharma (Rosenberg, Germany). Croscarmellose sodium (Ac-Di-Sol) was obtained from DuPont (Midland, MI, USA). Silicon dioxide (Aerosil 200) was acquired from Evonik Industries (Essen, Germany). Mannitol (Pearlitol 400 DC) was a kind gift from Roquette Pharma (Lestrem, France). Magnesium stearate was provided by Hungaropharma Ltd. (Budapest, Hungary). The water used in the experiments was from a Millipore Milli-Q ultrapure water system.

### Culturing of C. Butyricum

*C. butyricum* was grown in CNM at 37°C under anaerobic conditions for 48 h, which resulted in a mixture of spores and vegetative cells. *C. butyricum* was grown in TSB at 37°C under anaerobic conditions for 24 h, which resulted in exclusively vegetative bacteria. Anaerobic conditions were generated by using AnaeroGen anaerobic gas generating sachets in sealed jars. The cultures were centrifuged at 9000 rpm for 10 min. The spores/cells were then washed twice with phosphate-buffered saline (pH = 7.4) and resuspended in an appropriate volume of sterile water to obtain concentrations of bacteria from 10^7^ to 10^8^ colony-forming units (CFU)/mL.

### Microscopical Analysis

A laboratory light microscope (Olympus BH-2, Tokyo, Japan) was used at × 2000 magnification to study the bacterial cells and spores. Spore staining using malachite green was applied to differentiate the spores from the vegetative cells (40). Fibers were dissolved in purified water, and bacteria were centrifuged and washed before spore staining.

### Scaled-up Electrospinning of C. Butyricum

Electrospinning was carried out using a HSES setup comprising a spinneret (stainless steel, *d* = 34 mm) equipped with orifices (*d* = 330 μm) connected to a high-speed motor (21) (Fig. 1). HP-β-CD was added to the purified and resuspended bacterial cells/spores, and the mixtures were stirred at room temperature aerobically until full dissolution of the cyclodextrin (about 30 min). Electrospinning was carried out under aerobic conditions right after the preparation of the solutions. In each experiment, a SEP-10S Plus type syringe pump was used to feed 60 mL solution with 300 mL/h flow rate. The rotational speed of the spinning head was 40,000 rpm. The applied voltage was 40 kV (positive, direct current) (Unitronik Ltd., Nagykanizsa, Hungary). The dried material was removed from the interior of the drying chamber by air knives. A constant airflow (120 m^3^/h, room temperature, 40–45% relative humidity) was applied to help the dried fibers to reach the cyclone. The produced fibrous material was collected in a collection vessel connected to the cyclone. The electrospinning experiments were performed at ambient temperature (25°C) and under conditions of reduced risk for microbial contamination.

### Scanning Electron Microscopy

Sample morphology was studied by a scanning electron microscope (JEOL 6380LA, Tokyo, Japan) in high vacuum. Samples were sputtered by gold (~ 5–8-nm layer thickness) using a JEOL 1200 type ion sputter (Tokyo, Japan). 10–15 kV accelerating voltage and 10-mm working distance was used.

### Residual Water Content Measurement

A Q5000 TGA instrument (TA Instruments, New Castle, USA) was used to determine the residual water content of the samples (*n* = 3). Measurements were carried out under nitrogen atmosphere. A 10°C/min heating speed was used to heat up the samples from 25 to 105°C, which were kept at 105°C for 10 min. A 50 mL/min nitrogen flush was applied during the measurement.

### Viability Test

The viability of *C. butyricum* in the cultures, in HP-β-CD solution, and in HP-β-CD fibers (measured right after electrospinning) was determined by the CFU measurement method. Serial ten-fold dilutions were prepared from the samples in sterile water, and 100 μL was plated from each of the dilutions onto Columbia Blood Agar plates. The plates were incubated anaerobically at 37°C for 48 h. The plates with the appropriate number of colonies (10 to 200) were counted.

### Butyrate Production Measurement

Butyric acid production capacity of the bacteria in the fibers was tested by inoculating 50 mL CNM with 50 mg bacteria-containing electrospun material. The inoculated medium was incubated anaerobically at 37°C for 24 h. Butyric acid content in the samples was measured by reversed-phase high-performance liquid chromatography (RP-HPLC) (Agilent 1200 series LC System). An isocratic elution of water containing 0.5% phosphoric acid and ACN (85:15 V/V ratio) was performed at a flow rate of 1.0 mL min^−1^ and 25°C for 5 min. The UV detection wavelength was set to 210 nm. A 3 μL of sample volume (after 10× dilution) was injected onto a Phenomenex Luna C18 column (3 μm; 100 × 4.6 mm).

### Storage Stability Test

The prepared bacteria-containing fibers were kept in locked glass vials at − 20°C, 4°C, and room temperature aerobically and anaerobically. The viability of bacteria in the samples was assessed after 1 week, 1 month, 3 months, 6 months, and 12 months of storage. Anaerobic conditions were generated by using AnaeroGen anaerobic gas generating sachets in sealed jars.

### Grinding/Milling of the Fibers

An oscillating mill (Quick 2000 Kft., Tiszavasvári, Hungary) with a 1.0-mm sieve at 100 1/min was used to grind the bacteria-containing fibers to prepare a powder.

### Powder Characterization

An ERWEKA SVM12 tapped density tester (Heusenstamm, Germany) was used to measure bulk and tapped densities of the samples.

### Tableting

Direct compression tableting was carried out to prepare 600 mg round convex-shaped tablets from the ground bacteria-loaded electrospun fibers with an eccentric CPR-6 tablet press, equipped with 14-mm concave punches (Dott. Bonapace, Limbiate, Italy). The composition of the tablets was the following: 31.5% MCC, 31.5% mannitol, 10.0% croscarmellose sodium, 1.0% aerosil, 1.0% magnesium stearate, and 25.0% fibrous powder. The compression force was about 0.7 kN.

### Characterization of the Tablets

A Schleuniger 4M hardness tester (Thun, Switzerland) was used to measure the tablet breaking force of 5 tablets. Friability of 5 tablets was assessed by a Pharma Test PTF 20E (Hainburg, Germany) friability tester after 100 rounds.

### Statistical Analysis

Data are presented as average ± standard deviation (SD). Fiber diameter, bacteria viability, and long-term stability data were analyzed applying one-way analysis of variance with Tukey’s post hoc test (with 95% confidence level) using the Statistica 13.3 software (TIBCO Software, Palo Alto, USA).

## RESULTS AND DISCUSSION

### High-Speed Electrospinning of the Bacteria-Containing Cyclodextrin Solution

Cyclodextrins form strong aggregates in their concentrated solutions, and the intermolecular interactions make it possible to electrospin cyclodextrin solutions into fibers. Previous studies showed that hydroxypropyl-β-cyclodextrin fibers can be produced from aqueous solutions with 61.5 w/w% (single-needle electrospinning) (41) and 67.4 w/w% (HSES) (28) concentrations. Electrospinning of hydroxypropyl-β-cyclodextrin solutions containing sporulated and vegetative *C. butyricum* was attempted using the high-speed electrospinning device, and the fiber formation could be performed seamlessly. The runtime and the feeding rate were about 15 min and 300 mL/h in all of the experiments, respectively. Additional information on the experiments can be found in Table I. The electrospinning process yields were ~ 84% and ~ 48% for the samples incorporating sporulated and vegetative bacteria, respectively. Material loss was noticed on the wall of the drying chamber and in the pipes of the machine. The relatively low yield of the electrospinning of the vegetative sample might be caused by the presence of the residuals from the cell culturing medium. Supposedly, in the case of longer production, the material loss would not increase significantly, and therefore higher process yields (> 95%) could be achieved.

**Table I:** Production Data of the Scaled-Up High-Speed Electrospinning of Sporulated and Vegetative *C. butyricum*

	Sporulated sample (*n* = 3)	Vegetative sample (*n* = 3)
Cell state composition	30% spores 70% vegetative cells	100% vegetative cells
Feeding rate (mL/h)	300	300
Solution density (g/cm^3^)	1.197	1.194
Solid content of the solution (w/w%)	68.4	66.7
Yield (%)	83.8 ± 4.3	48.3 ± 9.5
Water content of the fibers (wt%)	7.3 ± 0.6	6.3 ± 0.2

**Fig. 2:**
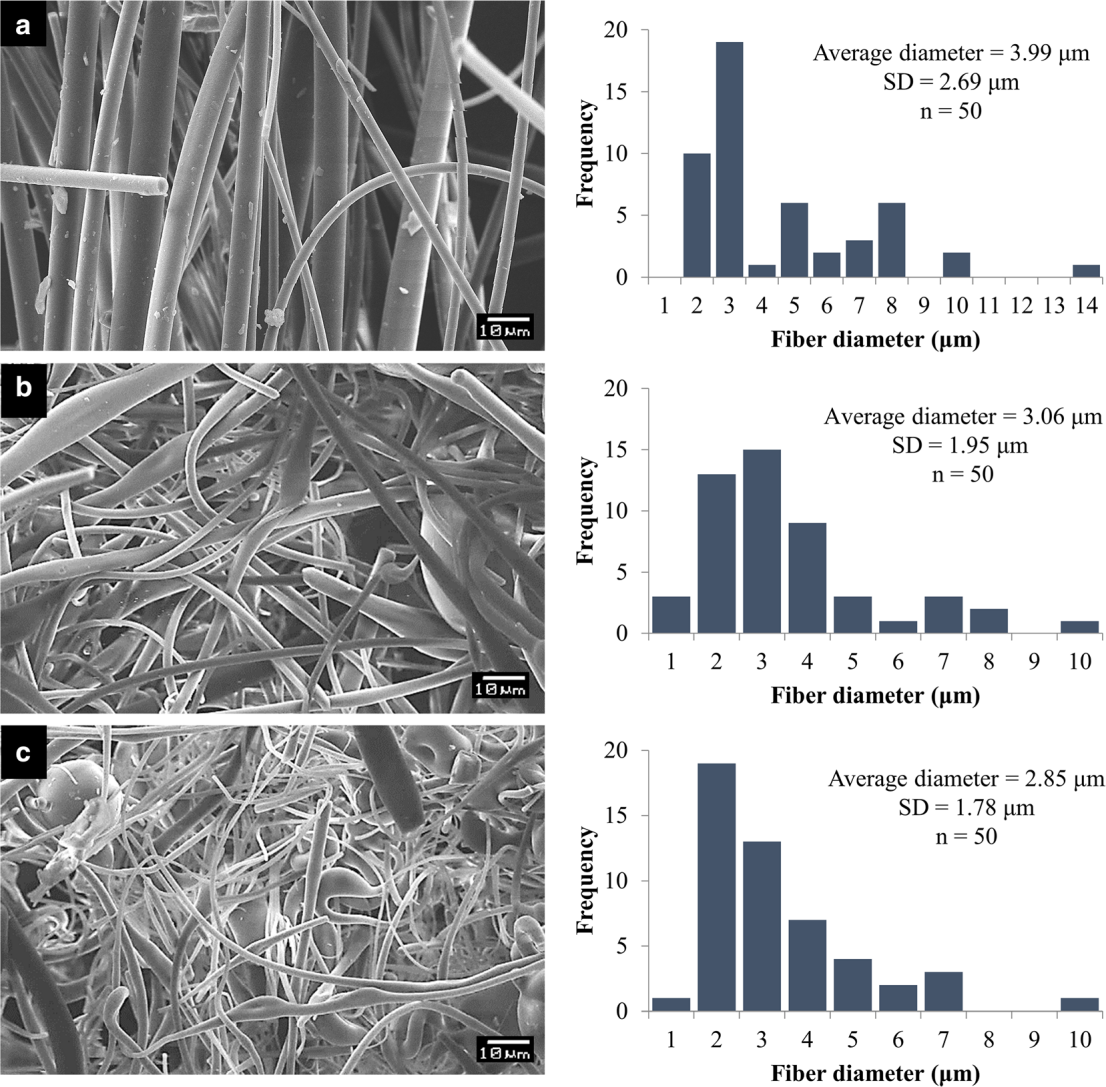
Scanning electron microscope images and histograms of fiber diameter distribution of HP-β-CD fibers without bacteria (**a**), sporulated (**b**), and vegetative (**c**) bacteria containing fibers prepared by HSES

Figure 2 shows SEM images of placebo (without bacteria) and bacteria-containing HP-β-CD fibers. The average fiber diameter of the placebo sample (3.99 ± 2.69 μm) was slightly larger than that of the bacteria containing samples (sporulated, 3.06 ± 1.95 μm; vegetative, 2.85 ± 1.78 μm). However, the difference was statistically significant only between the placebo and the vegetative sample (*p* < 0.05). Fiber diameter of the sporulated and vegetative bacteria-containing fibers did not differ significantly (*p* > 0.05). A slight difference in fiber morphology of the different samples could be observed: the bacteria-containing fibers seem to be a less homogenous and uniform compared with the placebo fibers. No vegetative cells or spores were visible on the outer surface of the fibers, which suggests that the bacteria were incorporated into the cyclodextrin fibers.

Figure 2 shows some fragmentation of the fibers, which could be accredited to the circular motion of the dried fibers in the collection container of the cyclone.

The residual moisture content of the fibers containing sporulated and vegetative bacteria was ~ 7.3 wt.% and ~ 6.3 wt.%, respectively, whereas the reference HP-β-CD powder was around 6.8 wt.%. This means that rapid and continuous drying of the fibers to the equilibrium moisture content of the matrix was possible at room temperature by HSES. The productivity of the technology with the applied feeding rate was around 150 g/h, attesting that more than 3.5 kg bacteria-containing electrospun material can be produced in a day by using high-speed electrospinning.

### Viability of Bacteria in the Cyclodextrin Solution and the Fibers after Electrospinning

**Fig. 3:**
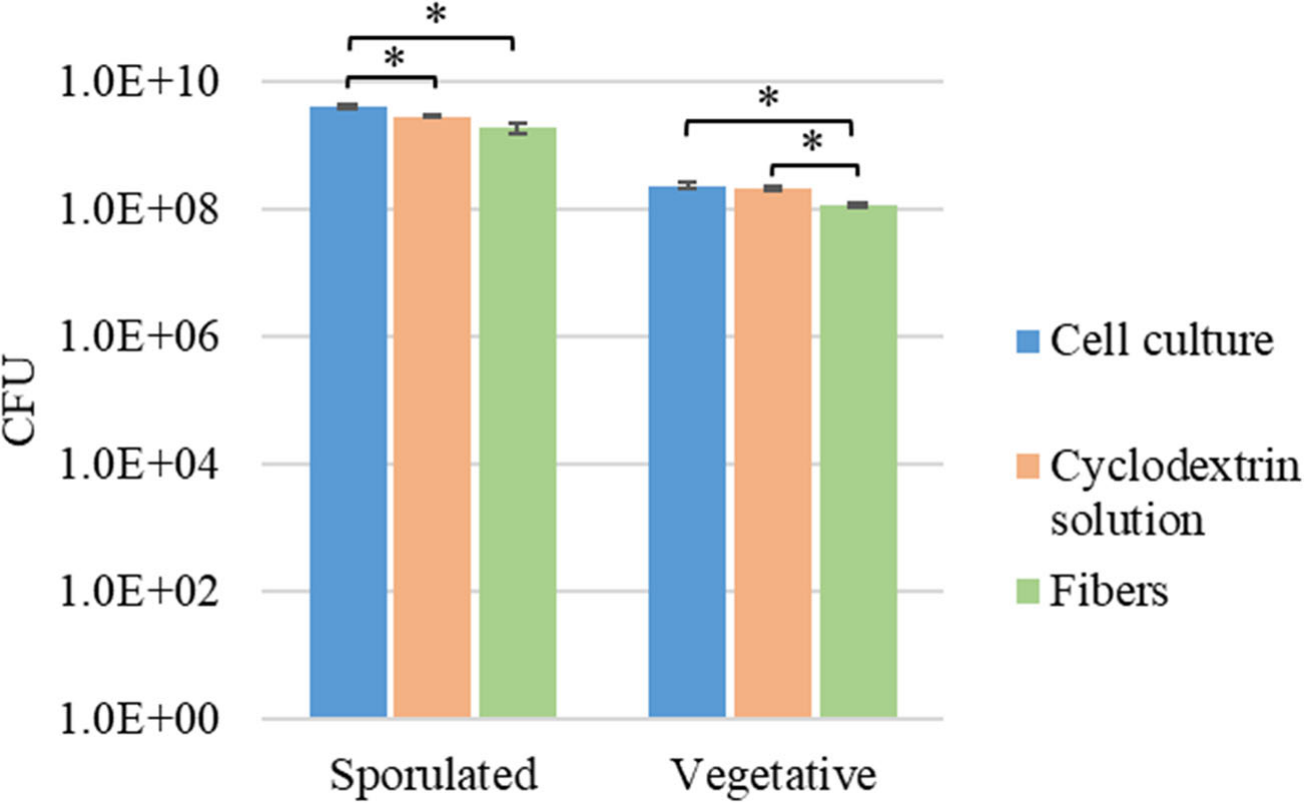
Survival of sporulated and vegetative *Clostridium butyricum* in the cyclodextrin solution and the electrospun fibers compared with the original cell culture viability (CFU, colony forming units; significant (*p* < 0.05) differences between groups are marked with *)

The effect of the cyclodextrin solution and the electrospinning process on *Clostridium butyricum* was evaluated by CFU measurements. The survival of sporulated and vegetative bacteria compared with the starting cell culture viability can be seen in Fig. 3. The number of viable cells or spores was reduced by a maximum of 0.16 log unit in the cyclodextrin solution and by a maximum of 0.33 log unit in the electrospun fibers. Vegetative cells and spores are exposed to changes in the osmotic environment when introduced into the cyclodextrin solution and high voltage and ultrafast solvent evaporation during the electrospinning process, which could presumably cause the observed slightly decreased viability.

Butyrate, produced by *C. butyricum*, has been shown to have anti-inflammatory effects, and therefore, it is important to investigate if the butyrate production capacity of the bacteria is retained after electrospinning. The medium was inoculated with bacteria-containing fibers, and the sample was checked for the presence of butyrate after 24 h of anaerobic incubation. The HPLC measurement showed the presence of butyrate in the samples (~ 1 μg/mL), besides the other medium components. The measurement confirmed that the ability of *C. butyricum* to produce this anti-inflammatory molecule was preserved in the fibers proving that this innovative solid form of the bacteria has real therapeutic potential.

### Long-Term Stability of the Bacteria in the Fibers

**Fig. 4:**
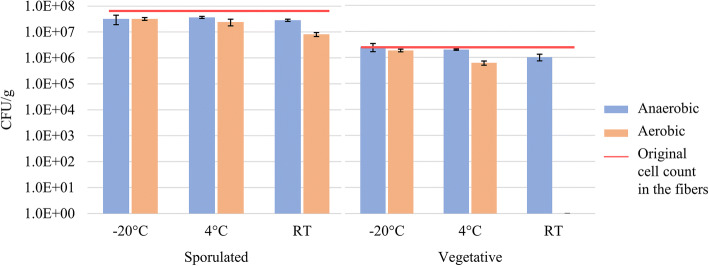
Stability of sporulated and vegetative *Clostridium butyricum* in the electrospun HP-β-CD fibers after 1 year of anaerobic and aerobic storage at − 20°C, 4°C, and room temperature

Long-term viability of bacteria in the fibers is crucial for the application in solid dosage forms. The viability of the fibrous samples containing sporulated and vegetative *Clostridium butyricum* stored at different temperatures aerobically and anaerobically can be seen in Fig. 4. It was observed that the samples stored anaerobically at 4°C and room temperature had higher viability compared with the samples stored in the presence of oxygen at the same storage temperature (*p* < 0.05). Survival rates of samples stored aerobically and anaerobically at − 20°C did not differ significantly. The lowest survival rates belonged to the samples stored at room temperature aerobically—the number of viable cells in the vegetative sample was reduced to 0. However, high viability of this strictly anaerobic bacterial strain could be preserved at room temperature in the presence of oxygen, as the viability loss of the sporulated sample was less than 1 log unit after 1 year. The outstanding stability at room temperature, aerobic storage (the preferred storage conditions from a practical perspective), suggests that the growth conditions used to prepare the sporulated bacteria were favorable for the long-term stability in the fibers compared with the growth conditions used to culture exclusively vegetative cells.

The homogeneity of bacteria in the fibers was assessed after 1 year by taking 3 samples from different locations of the fibrous mass. No significant differences were found in cell count between the different samples which indicates that living bacteria is homogeneously distributed in the fibrous mass.

The state of bacteria in the fibers was evaluated to see if drying by electrospinning induces sporulation of *C. butyricum*. Bacteria were spore stained before electrospinning and after 3 months and 1 year of storage of the fibers. The spore stained samples were observed under the microscope. The ratio of spores and cells was approximately the same before electrospinning, after electrospinning, and after storage (at all the different storage conditions), which suggests that drying by electrospinning and being encapsulated in HP-β-CD fibers for an extended time do not induce sporulation of *C. butyricum*.

### Milling of the Bacteria-Containing Fibers

**Fig. 5:**
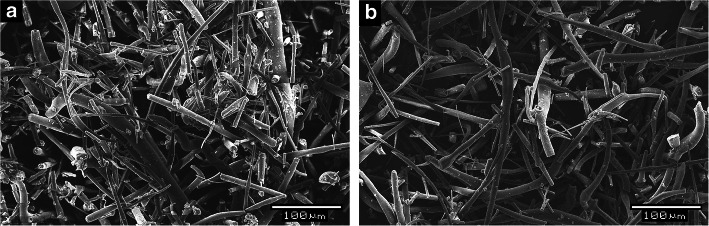
SEM images of the ground fibers containing sporulated (**a**) and vegetative (**b**) bacteria

Electrospun bacteria-loaded fibers need to be processable to be able to develop solid pharmaceutical products from the electrospun material. Even though fiber fragmentation was observed in the cyclone and in the collector bin, the bulk density and flowability of the fragmented bacteria-loaded fibers were not suitable for conventional tableting by direct compression. Therefore, the produced fibers had to be milled to gain a powder with improved flowability. Milling of the bacteria-loaded fibrous material was performed after the HSES process by an oscillating mill. There was no need for secondary drying of the bacteria-containing fibers, they had outstanding friability, and the result of the milling was a fibrous powder. The SEM image of the ground fibers shows that the fibers got broken into many smaller fragments with fibrous structure (Fig. 5). The fragment length of the sporulated and vegetative sample was 100.5 ± 85.0 μm and 105.6 ± 94.2 μm, respectively. The yield of the small-scale grinding was ~ 86%. The material that remained in the oscillating mill was not sticky, lumped, or molten.

### Mixing the Ground Fibers with Excipients, Powder Characterization, and Tableting

The ground fibers exhibited the properties of a light-density material (Table II) with poor flow properties. Therefore, excipients were mixed with the ground fibers to improve flowability and to gain a powder appropriate for tableting. Flowability properties of the powder blend containing the milled bacteria-containing cyclodextrin fibers and the excipients were superior compared with the flowability of the ground fibers (Table II). The obtained powder blend was suitable for tableting by direct compression.

**Table II:** Flowability Properties of the Powder Blend Containing the Bacteria-Loaded Fibers and Excipients

	Ground fibers	Ground fibers mixed with excipients
Bulk density	0.150 g/mL	0.450 g/mL
Tapped density	0.240 g/mL	0.628 g/mL
Hausner ratio	1.60	1.40
Carr index	33.3%	28.3%

Table III summarizes the results of the tablet characterization measurements. The tablets were found to be free from capping, chipping, and sticking.

**Table III:** Properties of the Prepared Tablets

Compression force	0.77 ± 0.18 kN
Individual weight	606.2 ± 12.6 mg
Tablet breaking force	89.2 ± 11.6 N
Friability	0.9%

### Bacteria Survival after Milling and Tableting of the Electrospun Fibers

**Fig. 6:**
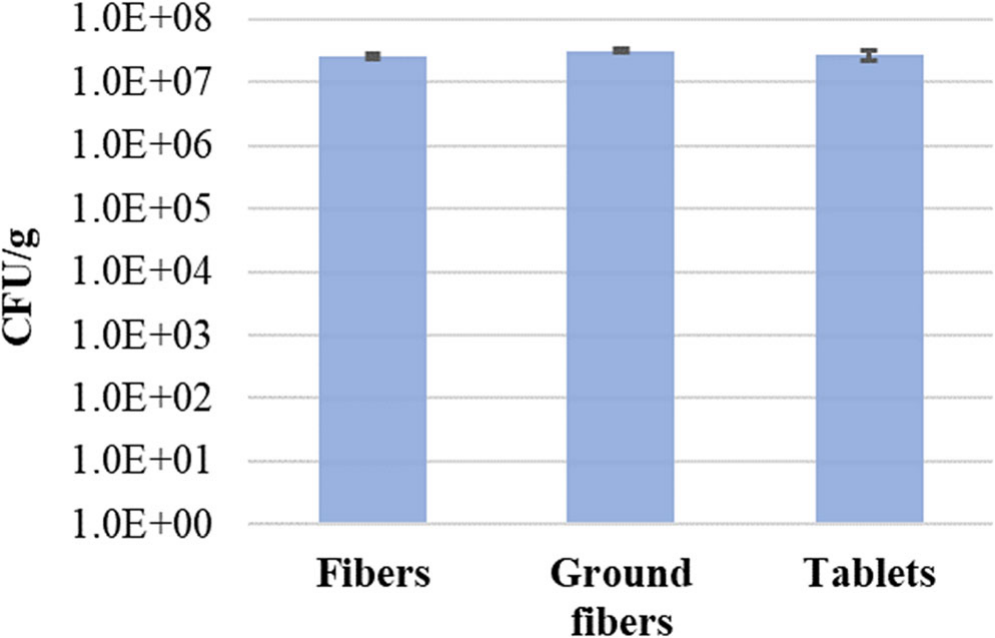
Bacteria survival after oscillating milling and tableting of the electrospun fibers

CFU measurements were carried out after milling and tableting to evaluate the effect of the fiber processing steps (milling and tableting) on the viability of *C. butyricum*. Figure 6 does not show significant bacterial viability loss (*p* > 0.05) in the fibers after the processing steps.

## CONCLUSION

The present work demonstrated that high-speed electrospinning is capable of gently and continuously producing processable fibers that incorporate anaerobic bacteria from the gut microbiome. Bacteria-containing fibers with a water-soluble cyclodextrin matrix (HP-β-CD) were prepared with 300 mL/h feeding rate, corresponding to ~ 150-g solid product per hour. The viability of the electrospun sporulated anaerobic bacteria was preserved after 1 year of aerobic storage at ambient temperature, which makes a flexible drug storage possible. The bacteria-containing fibers were grindable by an oscillating mill, and the milled fibrous powder could be tableted by direct compression after mixing with tableting excipients. None of the processing steps (drying, milling, and tableting) caused significant reduction in bacterial viability. The developed oral dosage form of the anaerobic gut bacteria strain, *Clostridium butyricum*, shows that electrospinning is a promising formulation technology for potential microbiome therapeutics.
